# Rationale and design: telepsychology service delivery for depressed elderly veterans

**DOI:** 10.1186/1745-6215-10-22

**Published:** 2009-04-20

**Authors:** Leonard E Egede, Christopher B Frueh, Lisa K Richardson, Ronald Acierno, Patrick D Mauldin, Rebecca G Knapp, Carl Lejuez

**Affiliations:** 1 Center for Disease Prevention and Health Interventions for Diverse Populations, Ralph H. Johnson VAMC, Charleston, South Carolina, USA; 2 Division of General Internal Medicine & Geriatrics, Medical University of South Carolina, Charleston, South Carolina, USA; 3 Center for Health Disparities Research, Medical University of South Carolina, Charleston, South Carolina, USA; 4 The Menninger Clinic and Department of Psychiatry, Baylor College of Medicine, Houston, Texas, USA; 5 Department of Psychiatry, Medical University of South Carolina, Charleston, South Carolina, USA; 6 School of Psychology, Murdoch University, Perth, Western Australia, Australia; 7 Department of Clinical Pharmacy and Outcome Sciences, Medical University of South Carolina, Charleston, South Carolina, USA; 8 Department of Biostatistics, Bioinformatics, and Epidemiology, Medical University of South Carolina, Charleston, South Carolina, USA; 9 Department of Psychology, University of Maryland, College Park, Maryland, USA

## Abstract

**Background:**

Older adults who live in rural areas experience significant disparities in health status and access to mental health care. "Telepsychology," (also referred to as "telepsychiatry," or "telemental health") represents a potential strategy towards addressing this longstanding problem. Older adults may benefit from telepsychology due to its: (1) utility to address existing problematic access to care for rural residents; (2) capacity to reduce stigma associated with traditional mental health care; and (3) utility to overcome significant age-related problems in ambulation and transportation. Moreover, preliminary evidence indicates that telepsychiatry programs are often less expensive for patients, and reduce travel time, travel costs, and time off from work. Thus, telepsychology may provide a cost-efficient solution to access-to-care problems in rural areas.

**Methods:**

We describe an ongoing four-year prospective, randomized clinical trial comparing the effectiveness of an empirically supported treatment for major depressive disorder, Behavioral Activation, delivered either via in-home videoconferencing technology ("Telepsychology") or traditional face-to-face services ("Same-Room"). Our hypothesis is that in-homeTelepsychology service delivery will be equally effective as the traditional mode (Same-Room). Two-hundred twenty-four (224) male and female elderly participants will be administered protocol-driven individual Behavioral Activation therapy for depression over an 8-week period; and subjects will be followed for 12-months to ascertain longer-term effects of the treatment on three outcomes domains: (1) clinical outcomes (symptom severity, social functioning); (2) process variables (patient satisfaction, treatment credibility, attendance, adherence, dropout); and (3) economic outcomes (cost and resource use).

**Discussion:**

Results from the proposed study will provide important insight into whether telepsychology service delivery is as effective as the traditional mode of service delivery, defined in terms of clinical, process, and economic outcomes, for elderly patients with depression residing in rural areas without adequate access to mental health services.

**Trial registration:**

National Institutes of Health Clinical Trials Registry (ClinicalTrials.gov identifier# NCT00324701).

## Background

People who live in rural areas experience significant disparities in health status and access to care compared to their urban counterparts [1,2]. Access to appropriate mental health care services represents a significant problem in many rural and remote areas and as the ageing population expands, this problem will intensify over the next several decades without innovative solutions [1-4]. There is growing awareness that specifically tailored treatment and service delivery strategies are frequently needed for different populations of adult consumers of mental health services [3,5]. Access to appropriate mental health care services represents a significant problem in many rural and remote areas and this problem will intensify over the next several decades without innovative solutions [4]. In the mental health field, "telepsychology," (or "telepsychiatry" or "telemental health") represents a strategy for potentially addressing this longstanding access to care problem [6-8].

The strengths of telemental health include well-documented patient and provider satisfaction for a range of services [9-15]; strong support for the reliability of clinical assessments (eg, neuropsychological testing, clinical interviews, and mental status exams) relative to face-to-face assessments [16-20]; and evidence that medical patients would choose to receive it if it was available and would improve their access to care [21]. In addition, research has documented the effectiveness of telepsychiatry to treat specific mental health diagnoses such as depression [22-24] and anxiety disorders [25-27], as well as its effectiveness with specific populations including incarcerated patients [28,29], children and adolescents [30], rural populations [13,31,32] and older adults [16,33-35]. The weaknesses identified in the research literature include a paucity of rigorous cost and efficacy studies, particularly for specific populations, and lack of research into the legal, ethical and regulatory issues inherent in this application of technology to clinical practice [6,7,36].

The use of telemedicine to address urgent patient care needs for older adults has been identified as the first two of five priority areas for future development by the Department of Veterans Affairs (VA) [37]. Although there is widespread use of telepsychology to provide clinical services within and outside of the VA [38], there is insufficient research to determine if telepsychology service delivery is as acceptable or effective as traditional service delivery for older adults. In addition it remains unknown whether it is cost-effective for elderly veterans with depression residing in rural areas, without access to specialty geropsychiatric services. Thus, research that addresses the efficacy of this mode of service delivery is urgently needed.

This paper describes the rationale, study aims and objectives, and research design and methods of an ongoing four-year prospective, randomized clinical trial comparing the effectiveness of Behavioral Activation treatment for geriatric major depression delivered via in-home videoconferencing technology ("Telepsychology") compared to traditional face-to-face delivery ("Same-Room"), to test the hypothesis that in-home "Telepsychology" service delivery will be equally effective as the traditional mode (Same-Room).

### Rationale

The population of older adults is particularly well-suited for telepsychology for several reasons: (1) Over the next two decades there will be a large increase in the rural elderly population, and access to care is already a significant problem for many elderly adults with major depressive disorder (MDD) [3]; (2) telepsychology may be preferred by older adults, who avoid traditional mental health care due to embarrassment or stigma [39-43]; and (3) telepsychology may address significant age-related problems in ambulation and transportation [3,44]. Indeed, medically disabled older adults with ambulation difficulties, and those isolated older adults in rural areas, are among those at greatest risk of psychopathology, yet currently are the least likely to be able to obtain treatment [3]. Finally, results from a recent randomized trial also found telepsychiatry to be efficacious in the treatment of mild cognitive deficits among elderly persons [34].

### Study Aims & Objectives

The objective of the study is to test the hypothesis that in-home video-conferencing technology ("Telepsychology") will be as effective as the more traditional mode of service delivery ("Same-Room") for treating older adult veterans suffering from major depressive disorder (MDD). This objective will be accomplished by (1) Using an established, highly effective and well-tolerated behavioral treatment for depression (i.e. Behavioral Activation Treatment for Depression (BATD) [45-47]; (2) Employing a randomized between groups experimental design; (3) Examining standardized, repeated dependent measures of: (a) clinical outcomes, such as symptom severity, social functioning, and medical status; (b) process variables, such as patient satisfaction, treatment adherence, and treatment dropout rates; and (c) economic outcomes; and (4) conducting analyses to examine for outcome differences between African-American and Caucasian patients.

The primary research questions of the proposed study are:

1. Is in-home telepsychology for major depressive disorder in older adults as effective as same-room treatment on clinical and process outcomes?

2. Is in-home telepsychology for major depressive disorder in older adults cost-effective?

## Methods

The study is a 2 group randomized controlled trial with randomization of individual participants, blinded outcomes assessments at baseline, mid-treatment (4-weeks), post-treatment (8-weeks), 3-months, and 12-months, and concurrent economic evaluation.

### Location and Setting

The assessments and interventions will be conducted at the primary care and mental health clinics of the Ralph H. Johnson Veterans Affairs Medical Center (VAMC) located in downtown Charleston, South Carolina. The Ralph H. Johnson VAMC is a primary, secondary, and tertiary referral medical center providing acute medical, surgical, and psychiatry inpatient care as well as primary care and specialized outpatient services. Currently, there are 98 acute care beds and 28 nursing home care unit beds for a total of 126 operational beds. The primary service area extends from north of Myrtle Beach, SC, down to the surrounding counties of Savannah (Georgia, USA). For the most recent fiscal year (FY 2007), there were 41,645 unique patients, 4,000 discharges, 475,714 total outpatient visits, and 2,968 OEF/OIF enrollees and users. The Ralph H. Johnson VA Medical Center is a part of VA Southeast Network VISN 7 which includes facilities in Charleston, SC, Columbia, SC, Atlanta, GA, Augusta, GA, Dublin, GA, Birmingham, AL, Tuscaloosa, AL, and Central Alabama (Montgomery and Tuskegee campuses). The facility also has four Community Based Outpatient Clinics (CBOCs) in (1) Beaufort, SC – 53 miles to VAMC; (2) Goose Creek, SC – 17 miles to VAMC; (3) Myrtle Beach, SC – 83 miles to VAMC; and (4) Savannah, GA – 84 miles to VAMC.

### Ethics and Trial Registration

The study is funded by grant #IIR 04-421 from the Veterans Health Administration Health Services Research and Development program. The trial is approved by the joint Institutional Review Board (IRB) of the Ralph H. Johnson VAMC and the Medical University of South Carolina (HR#16402). The trial is registered on the United States National Institutes of Health Clinical Trials Registry (ClinicalTrials.gov identifier# NCT00324701), available online at: 

### Trial Population and Recruitment

Subjects will be 224 older male and female veterans (age 60 or older) presenting for services at VA primary care clinics and meeting DSM-IV [48] criteria for MDD. Actively psychotic or demented persons, individuals with both suicidal ideation and clear intent, and individuals meeting criteria for substance dependence will be excluded from participation; however, in order to maximize generalization of results, presence of other forms of psychopathology (eg, anxiety disorders) will not be a basis for exclusion. Participants who are incapable of giving informed consent due to mental incapacity or extreme distress will be excluded from the study. Because the inclusion of minorities in research on psychiatric disorders is important, about 40% of our sample will be African American, which is representative of the population of South Carolina.

We will use two complementary approaches to identify eligible study subjects. The first method will consist of systematic identification of patients with MDD. After obtaining IRB approval for a partial waiver of HIPAA, we will use clinic-billing records over the previous 12-month period to identify subjects with ICD-9 codes consistent with a diagnosis of MDD. The physicians of eligible patients will be notified of their patients' potential eligibility and asked permission to enroll their patients in this study. After consent is obtained from the physicians, letters of invitation on clinic letterhead signed by the patient's physician will be mailed to patients from the study clinics. The letter will provide information about the study, explain the study requirements, and clarify that only subjects that meet certain criteria will be eligible to participate in the study. The letter will include an addressed and stamped post-card that subjects can mail back to indicate interest or lack of interest in participating in the study. In addition, the letter will provide a telephone number that interested subjects can call to receive detailed information about the study. In the letter, subjects will also be informed that they will receive a follow-up call in two weeks unless they mail back the post card or call to decline being contacted. Subjects that mail back the post card and express interest or call the provided telephone number will receive detailed information about the study. Subjects who agree to participate will be asked to provide written consent and will be scheduled for the initial screening assessment.

The second method will consist of referrals from physicians, other clinic staff such as nurses, or patients themselves in response to recruitment flyers for the study. The Principal Investigator (PI) will share the goals of the study and inclusion/exclusion criteria with physicians and clinic staff during clinic administrative meetings. Physicians and clinic staff will be asked to refer appropriate subjects to the study research assistants. In addition, IRB approved recruitment flyers will be posted in prominent locations in the study clinics.

Regardless of recruitment pathway, research staff will obtain written informed consent, complete a screening for dementia and psychosis, and assure that participants meet DSM-IV [48] criteria for MDD. Those candidates meeting MDD criteria will then complete the remainder of the assessment battery (Tables 1 and 2).

**Table 1:** Data Collection Schedule

Study Instrument	Screening Visit	Baseline Visit	4 Weeks Visit	8 Weeks Visit	3 Months Visit	12 Months Visit
**Screening/Baseline Assessment**						

Demographic Questionnaire	X					

Geriatric Depression Scale	X	X	X	X	X	X

SCID		X				X

Short Portable Mental Status Questionnaire	X					

						

**Multi-Method Assessment**						

Beck's Depression Inventory		X	X	X	X	X

Beck's Anxiety Inventory		X	X	X	X	X

SF-36		X	X	X	X	X

Medical Outcomes Study Social Support Form		X			X	X

Morisky Medication Adherence Form		X			X	X

						

**Process Variables**						

Charleston Psychiatric Satisfaction Scale			X	X	X	X

Treatment Credibility			X	X	X	X

Service Delivery Perceptions			X	X	X	X

Treatment Adherence (Therapists)			X	X	X	X

Session Attendance/Attrition (Therapists)			X	X	X	X

Prior Computer/Audiovisual Tech. Experience			X	X	X	X

						

**Resource Utilization**						

Baseline Visit Form		X				

Standard Follow-up Form					X	X

**Table 2:** Study Instruments

**Geriatric Depression Scale** **(GDS) **[51]	The GDS is one of the most widely used measures of depression in the elderly population using the generally accepted cutoff score of 11. This 30-item measure shows good test-retest reliability and internal consistency. The GDS exhibits good concurrent validity, and excellent sensitivity, specificity, and positive predictive power in assessing depression with older adults.
**The Short Portable Mental Status Questionnaire **[85]	This is a ten item test that is quickly completed and will be used to screen for cognitive impairment (cutoff ≥ 7). The screen is effective in identifying cognitive impairment in a variety of geriatric populations.

**Structured Clinical Interview for DSM-IV** **(SCID-IV) **[50]	MDD and other psychopathology will be evaluated using this structured clinical interview based on the DSM-IV. The onset of the MDD will be specified. The SCID-IV has excellent interrater reliability on assessments of symptoms across a variety of disorders (overall kappa = 0.85).

**Beck Depression Inventory** **(BDI) **[52]	The BDI is a 21-item self-report scale, is among the most widely used instruments to measure depression. The BDI has high internal consistency (α = 0.86 – 0.91).

**Beck Anxiety Inventory** **(BAI) **[66]	The BAI is a 21-item self rating scale of anxiety symptomatology. Specific symptom clusters have been identified reflecting neurophysiological, subjective, panic, and autonomic dimensions. The BAI has good internal consistency and concurrent validity with the Hamilton Anxiety Rating Scale.

**Medical Outcome Study Short Form-36 Health Survey (SF-36) **[67]	The SF-36 is a 36-item questionnaire that measures health status and functioning over the past four weeks. The items vary from dichotomous (yes/no) responses, to ratings on a 6-point Likert scale. Responses are compiled into eight dimensions covering (a) Functional Status; (b) Well-Being; and (c) Overall Evaluation of Health. The SF-36 has good test-retest reliability as well as sensitivity to change in health status.

**Medical Outcomes Study (MOS) Social Support Survey **[86]	It measures four functional components of social support: 1) tangible support; 2) affection; 3) positive social interaction; and 4) emotional or informational support. The total scale (α = 0.97) and subscales (α = 0.91 to 0.96) have high internal consistency, good criterion and discriminant validity, and one-year test-retest reliability (0.72 to 0.76).

**The Morisky Adherence Score **[87]	This is a commonly used self-report tool to assess adherence to medications. It has good validity and reliability. This scale asks patients to respond "yes" or "no" to a set of 4 questions. A positive response to any question indicates a problem with adherence with a total possible score of 4; higher scores indicate poorer adherence.

**Charleston Psychiatric Outpatient Satisfaction Scale (CPOSS) **[53]	The CPOSS is 16-item measure, with a Likert scale response format, based on a general measure of patient satisfaction. In a sample of patients preliminary data showed the measure had excellent reliability (alpha = 0.96) and good convergent validity with relevant anchor items ("would you recommend this treatment to a friend or family member?").

**Treatment Credibility Questionnaire **[88]	To assess for differences in outcome expectancy, treatment credibility scales developed by Borkovec and Nau (1972) will be used. Four of the questions will be used for this study, with 10-point Likert scales. These include questions regarding how logical the treatment seems, how confident participants are about treatment, and their expectancy of success.

**Service Delivery Perceptions Questionnaire**	This questionnaire will be used to assess subjects' perceptions about variables specifically related to the mode of service delivery (e.g., the quality of communication, ease of use, willingness to use treatment).

**Prior Experience with Computer and Audiovisual Technology Questionnaire**	We will administer a short measure to learn more about participants' prior experiences and comfort level with computers and audiovisual technology.

**Resource Utilization Questionnaire **[89]	Previously validated questions on resource utilization will be administered as part of the baseline, 3-, and 12 month assessments. The questionnaires are 1 page long and capture information on hospitalizations, physician/professional visits, and medications. The baseline assessment will capture differences between groups and allow for the control of possible group variation during data analysis.

### Randomization

**Figure 1:**
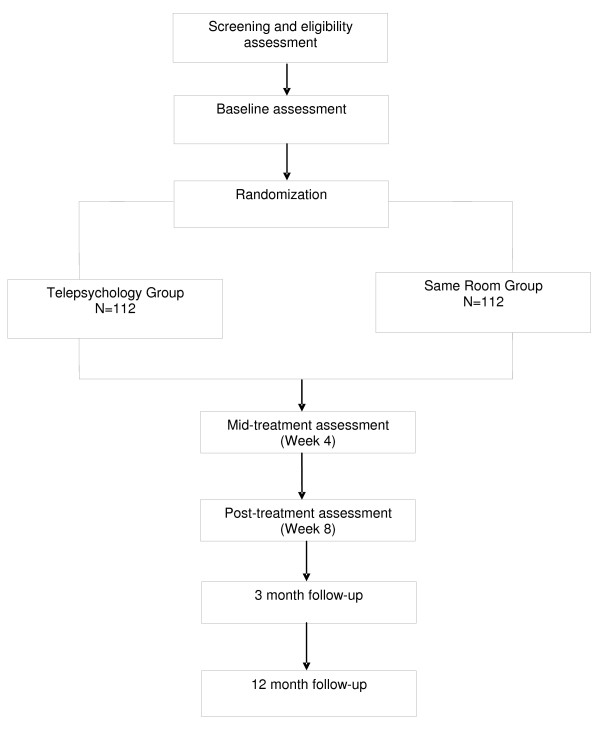
**Design and Study Flow**.

The study coordinator will verify all eligibility criteria prior to randomization. The procedure and risks will be explained to the patient and the consent form signed as per standard clinical practice. All subjects will be randomly assigned to one of two groups – Telepsychology (n = 112) and Same Room (n = 112). The randomization sequence will be generated by the senior biostatistician and provided in individual sealed envelopes to the data/statistical analyst who will be responsible for randomization. This information will remain confidential and not shared with the study sites in accordance with the CONSORT guidelines [49]. The study coordinator will call the data/statistical analyst to get subject randomization assignment. Once a randomization assignment is provided, the patient is entered into the study and will be included in the intention to treat analysis (Figure 1).

### Intervention and Control Groups

All subjects will receive the same individual mental health intervention for an 8-week period. Sessions will occur approximately once each week during this period, and each session will last for about 60 minutes. The intervention group (Telepsychology) will receive therapy via videophone while the control group (Same Room) will receive face-to-face therapy. All patients will receive 8 sessions of BATD. One of the strengths of the BATD protocol is that it is designed to be very straightforward, easily learned by therapists and patients, and can be implemented over an 8-session period. Initial sessions are used to build patient rapport and provide a rationale for activation-based treatment. Subsequent sessions are used to assess factors that might be maintaining depressed behavior (eg, determining if a deficit of reinforcing activities or a preponderance of punishing activities exists); assess functional aspects of the behavior itself (eg, is the depressed person receiving significant social reinforcement for being depressed); initiate efforts to increase the likelihood of engaging in reinforcing behaviors; and reduce the availability of unintended reinforcement for depressed behavior. Next, a systematic approach to behavioral activation is implemented. This is simply a process to increase the rate of enjoyable (ie, going to dinner with a friend) and functional (ie, grocery shopping) behaviors, with this increase resulting in a decrease in depression. Using a daily calendar, patients begin with a weekly self-monitoring exercise that serves as a baseline assessment of daily activities, and orients them to the quality and quantity of his or her activities on a daily basis. Following the introductory and assessment stages of BATD, emphasis in sessions two and three shifts towards the identification of goals within key life areas including relationships, employment, recreational activities, physical/health issues, and spirituality. From these goals, the individual will select specific behaviors that will help achieve these objectives. For example, the life area of family relationships may include the goal of rebuilding a relationship with a particular family member, which can be accomplished with specific behaviors such as calling the family member twice per week. To help facilitate engagement in the desired behaviors, each activity is placed within an activity hierarchy and rank-ordered from "easiest" to "most difficult" to accomplish. Patients then use a daily calendar to plan, *in advance*, the next week wherein they schedule a variety of activities, including both 'fun' (such as something they enjoy doing) or 'functional' (such as getting some undesirable chore out of the way so they do not have to worry about it any longer). The planning calendar serves as an activity log (in instances where patients do not follow the planned activity, they are instructed to update the calendar to reflect what they actually did). Behavioral Checkouts, or specific reviews of completed activities and concurrent moods are completed with therapists in session to monitor progress over the course of treatment as the patient graduates to different levels of the hierarchy of activities.

The therapist and patient collaboratively ensure that the behaviors that they are planning fit well within the life area and goals in that area as previously established. At the beginning of each session, the behavioral checkout occurs in which adherence to the planned calendar of activities is reviewed, and ensuing mood states are discussed. Specific behaviors and contextual parameters, including time of occurrence and duration of activity, are determined on the basis of the patient's level of success or difficulty with activities for the prior week. As an additional incentive for patients to complete the behaviors on the behavioral checkouts, patients are asked to identify and schedule weekly rewards, arranged to become available only after they have completed the planned activities a particular week. The treatment is flexible and we ensure that all activities (eg, pleasant activities lists) are age, gender, and culture appropriate.

Upon completion of the active treatment program described above, subjects from both conditions (Telepsychology, Same-Room) will be followed for 12 months. The outcome assessment battery will be administered to all subjects at the end of this follow-up period. Those who develop acute problems during follow-up will either be provided additional treatment by the project staff or will be referred to other appropriate treatment providers. In order to prevent distortions in the follow-up data, information on patients who receive further treatment will be analyzed separately.

### Telecommunications Technology

**Figure 2:**
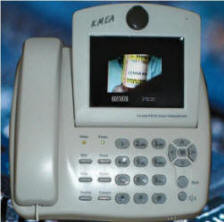
**Videophone**.

Treatment sessions for the Telepsychology condition will be conducted using in-home videoconferencing technology. We will use an analogue videophone (KMEA TV500SP, Figure 2) that operates via plain old telephone service (POTS). Apart from the video screen, this equipment appears and functions much like a basic touch-tone telephone. It is a "plug-and-use" product, with built-in camera, full duplex speakerphone, 4-inch LCD color screen (270 K pixels) with real-time motion display (18 frames per second), and oversized touch-tone buttons for easy use by all patients. This equipment is simple to use. If necessary, project or clinic staff will be available to visit participants in their homes to help set up the equipment. We will track the type and amount of assistance required across sessions in order to help anticipate future difficulties with in-home use.

### Study Instruments and Data Collection Schedule

Self-report measures will be used to evaluate clinical outcome at baseline, mid-treatment (4-weeks), post-treatment (8-weeks), 3-months, and 12-month follow-up. Process outcomes will be evaluated at 4-weeks, 8-weeks, 3-months, and 12-months. Resource Utilization surveys will be administered at baseline, and then 3 and 12 month follow-up. Endpoint psychiatric interviews (ie, Structured Clinical Interview for DSM-IV, [SCID]) [50]; will be administered at baseline and 12-months by raters blind to subject condition. Structured interviews will be audiotaped, and 20% will be rated by an independent to ensure interrater reliability. The complete assessment takes about 120 minutes. Study assessments will be administered by either the Research Coordinator. Assessment clinicians will not deliver treatment. Their training will include instruction, assigned readings, modeling, role-playing, and feedback.

### Therapist Training and Supervision

The same counselors will conduct the individual BATD treatment for both conditions (Telepsychology, Same-Room). At the start of the project, treatment providers will undergo a 12-hour training program with the clinical psychologist/project consultant and the developer of the BATD treatment manual. During the project, therapists will follow the BATD treatment manual, and will undergo weekly peer supervision with an experienced clinical psychologist on the team. Thus, a highly experienced and expert team of mental health care providers will implement the treatment for the subjects in this study. We have used a similar training approach in our other studies.

### Treatment Integrity and Adherence

A quantitative measure of protocol adherence will be obtained using a checklist of the specific procedures scheduled to be followed in the BATD treatment manual. To ensure treatment adherence, all sessions will be audiotaped, and 20% of these will be rated for competence and adherence by co-investigators. To evaluate adherence to treatment protocol, rating forms that use a 7-point Likert scale were developed and assess how well the therapists accomplished relevant behavioral tasks for each session. Two raters, blind to treatment condition, will rate tapes independently to allow for computation of inter-rater reliability. Our rating forms are modeled after the therapist rating forms used in other studies of cognitive-behavioral treatments, including our own treatment outcome studies that successfully demonstrated therapist fidelity to a manualized cognitive behavioral intervention delivered via "telepsychology" [27]. This will allow us to study any differences between conditions on non-specific factors, such as therapist empathy and rapport.

### Outcome Measures

#### Clinical Outcome Measures

The primary clinical outcome measure is the proportion (%) of patients who respond to treatment. Treatment Responder status will be determined using each of the dependent measures of depression (ie, Geriatric Depression Scale (GDS) [51]; Beck Depression Inventory (BDI) [52]; and SCID [50]. For the BDI and GDS, treatment responders will be those that demonstrate, at 12-month follow-up, improvement of at least 50% on the total score. For the SCID, treatment responders will be those who are no longer diagnosed with MDD. Dichotomous classifications for the GDS and the BDI will be achieved by defining treatment response as at least a 50% improvement from baseline to post-treatment level (percent change from baseline: [baseline-post]/baseline ≥ 50%). We will report analyses involving each measure of treatment response separately, but will take the a priori position that, in the case where results based on each of these analyses do not converge, overall conclusions regarding treatment response will be based on the two measures that concur. Thus, our indicator of treatment response will be in terms of each measure, as well as in terms of the converging data from at least two different measures.

The primary process outcome is score on the Charleston Psychiatric Outpatient Satisfaction Scale (CPOSS), [53] measured at the end of the 12-month follow-up period. The CPOSS is a 16-item measure with each item having a Likert-type format (total score is sum of the 16 items with range 16–80). Non-inferiority of patient satisfaction at the end of the 12-month follow-up period for the two modes of delivery will be evaluated via the one-sided non-inferiority 90% confidence interval approach to estimate the difference in end of study mean scores on the CPOSS for the Telepsychology and Same-Room conditions. The magnitude of the difference in satisfaction levels, as estimated by the confidence interval will provide useful clinical information and will allow a clinical judgment relative to the clinical non-inferiority of the of the two modes of delivery.

#### Economic Outcome Measures

The economic analysis will determine the cost and potential cost savings of Telepsychology. Comprehensive information will be gathered on 1) telepsychology-related capital expenditures, and 2) patient-related resource utilization over 12 months for all patients in the project. This will allow us to estimate the combined spectrum of investment expenditures and direct in-hospital cost of Telepsychology as well as cumulative health costs over 12 months. In addition, a Markov-type economic model [54], determining transition probabilities between different health states, will be developed to predict flow of funds and event rates over a 12-month period for Telepsychology participants based on outcomes.

### Sample Size Determination and Power Analysis

The primary response variable for sample size calculation is the proportion (%) of patients who respond to treatment. Treatment response is defined as at least a 50% improvement from baseline to post-treatment level on the GDS [percent change from baseline: (baseline-post)/baseline ≥ 50%]. Demonstration of non-inferiority among treatments differs from that of establishing superiority. For a non-inferiority trial, the null hypothesis states that two treatments are non-equivalent, that is they differ by at least a clinically relevant amount, Δ. [55-62]. The approach will be formulated in terms of the one-sided non-inferiority problem [55,60,61,63] because it is of interest only to test that the treatment response (% responders) for the Telepsychology mode of delivery is not too low compared to that for the standard Same-Room mode of delivery. Note that, for non-inferiority trials, the consequence of a false positive (ie, claiming the novel therapy [Telepsychology] is worse than the standard therapy [Same-Room] when it is, in fact, non-inferior) is to keep patients on the standard therapy. Therefore, the Type I error rate, α, can be set higher than in traditional superiority trials (eg 0.1–0.2) [64].

We estimate that the % treatment responder for Same-Room delivery (P) is 0.70. We assume that a maximum clinically unimportant difference in response proportion (Δ, the non-inferiority effect size) is 0.15 between Telepsychology and the standard Same-Room service delivery (upper limit of one-sided 90% confidence interval must not be greater than Δ = 0.15 for the conditions to be declared equivalent). Using the tables of Machin and Campbell [64] and assuming Δ = 0.15, one-sided α = 0.10, P = 0.70, and power = 0.85, we estimate that approximately n_1 _= n_2 _= 100 subjects per condition are needed for overall treatment effects comparisons. If we further assume that 10% of subjects will not have at least one post baseline measurement (i.e. will not be included in the ITT sample), then 112 subjects must be randomized to each treatment group to yield the required sample size for the primary intent-to-treat analyses (n_1 _= n_2 _= 100/group).

For the ITT sample (n_1 _= n_2 _= 100/treatment group) we will be able to estimate the individual (main effects) end of treatment means for each of the continuous clinical and process measures with a precision of ± 0.2 standard deviations. For the primary outcome measure Δ_Δ1 _= Δ_(Post)T_- Δ_(Post)S_, which measures the difference in change from baseline (pre to immediate post treatment) for the continuous outcome domain measures for the telepsychology condition compared to change from baseline for the standard (same-room) condition, we will be able to estimate with a precision of ± 0.3 standard deviation units using 95% confidence intervals for the difference in two means (Δ_(Post)T_, Δ_(Post)S_), where the appropriate standard deviation is the pooled standard deviation for comparing change from baseline for the telepsychology condition versus the standard condition.

Assuming that 20% of subjects will drop out of the ITT sample (n_1 _= n_2 _= 80, assuming equal treatment distribution of dropouts), we will be able to estimate for the completer sample the individual end of treatment means for each of the measures with a precision of ± 0.2 standard deviations, and the difference in change from baseline (pre to immediate post treatment) for the outcome domain measures with a precision of ± 0.3 standard deviation units.

Both the standard deviation and the distribution of costs are presently unknown for the type of patients who will be enrolled in Telepsychology. Thus, it is difficult to obtain a meaningful estimate of the power to detect a difference in CE from any arbitrary value. We propose to determine costs and apply to our economic model after the completion of the study, and then use the bootstrap technique to obtain a 90% Confidence Interval (CI) around the estimated costs. The bootstrap technique is appropriate because, as noted, the distribution of costs for these types of patients is presently unknown.

### Data Analysis

#### Primary Clinical Outcome

Testing the non-inferiority of the Telepsychology mode of delivery to the standard Same-Room mode in terms of % responders can be carried out through either a confidence interval approach or through a modified hypothesis testing procedure [55-62]. For both approaches, a conclusion that the two modes of delivery are similar in clinical efficacy (non-inferior) requires that the response proportion (% responders) for the standard delivery (Same-Room) cannot exceed by more than Δ the response proportion for the novel delivery (Telepsychology). We assume Δ = 0.15 between the novel delivery and the standard delivery (one-sided non-inferiority). With the confidence interval approach, the upper limit of the one-sided 90% confidence limit for the difference in % responders for the novel delivery (Telepsychology) and the standard delivery (Same-Room) must be 0.15 (Δ) or less to accept the hypothesis of a non-inferior novel treatment. Statistically, this translates into testing a non-zero difference between the treatments, where noninferiority is established by demonstrating that the difference between treatments is less than the maximum clinically unimportant difference in response proportions, Δ[55-57,59,61,62]. The hypothesis testing approach to evaluating equivalency in % responders, with an appropriately adjusted test statistic as described by Dunnett and Gent [56] and Farrington and Manning [57] will also be used.

In addition to comparing unadjusted % responders between the modes of delivery conditions, it also is of interest to compare % responders, adjusted for putative confounding variables. Possible covariables to be considered for inclusion in these analyses include initial disease severity as measured by baseline GDS or BDI score, use of psychiatric medication, number of other co-morbid conditions, and relevant others as identified though the preliminary descriptive analyses. Two approaches will be used to evaluate non-inferiority of adjusted % responders for the two delivery conditions. The method of Gart and Nam [65] and Nam [59], which involves calculation of a non-inferiority test statistic within strata of potential confounding variables, formulates a score test for a null hypothesis of a common specified clinically relevant non-zero difference in % responders between two treatments for multiple 2 × 2 tables. Because the above procedure may be limited by the number of covariables (number of strata) that can be used, we will carry out a second set of analyses using a multivariable logistic regression model to obtain estimates of adjusted % responding for each treatment delivery condition for a specified set of values for the covariables, and then will apply the methods described above (one-sided confidence intervals and non-inferiority hypothesis testing) to evaluate non-inferiority of the two interventions. The response variable for these analyses is the dichotomous outcome, responder/non-responder to treatment, with missing data from premature exits treated as non-responders. The primary independent variable is treatment condition.

#### Secondary Analyses

The one-sided non-inferiority 90% confidence interval approach (as described above) to evaluate noninferiority using the difference in change from baseline mean scores for the continuous clinical measures; GDS [51], BDI [52], BAI [66], SF-36 [67]; for the Telepsychology and Same-Room conditions will be used. The magnitude of the difference in the means (effect sizes), as estimated by the confidence intervals will provide useful clinical information and will allow a clinical judgment relative to the clinical non-inferiority of the two modes of delivery. The hypothesis testing approach to evaluating noninferiority, as described by Dunnett and Gent [56] and Farrington and Manning [57], will also be used.

#### Additional Analyses

In additional analyses, using the intent-to-treat sample and the baseline (week 0), mid- (week 4) and post- (week 8) active-treatment (randomized) phase values for continuous clinical variables (GDS, BAI, BDI, SF-36, patient ratings, functional status indicators), we will use random regression models (RRM) analyses (or equivalently, hierarchical linear model [HLM] or mixed effects models) to compare the Telepsychology and Same-Room conditions over the 8-week active phase time tracjectory. These analyses will use scores for each outcome as the dependent variable and delivery mode (treatment) and time (time by treatment) as the primary independent variables. The random regression approach estimates individual change in outcome for each subject in addition to estimating average change in outcome within each treatment population. Specifically, random regression models allow for measurement of subjects at different time points, missing data, and time varying or invariant covariates [68-74]. Further, longitudinal methods for binary outcomes [75-77] will be used to compare the Telepsychology and Same-Room conditions for the dichotomous outcome measure (MDD response status measured at weeks 4 and 8). Methods for categorical or ordinal outcomes [78-82] will also be applied where appropriate.

Exploratory multivariable logistic regression, assuming drop-outs are nonresponders, will be used to determine the set of predictor variables associated with treatment response (dichotomous outcome: treatment responder/nonresponder as described under primary analyses). In additional exploratory analyses, RRM analysis will be used to evaluate the effect of putative confounding or prognostic variables on continuous or binary clinical outcomes following the 8-week treatment course, and the possible effect modification (interaction) of these variables on the relationship between treatment status (delivery mode) and clinical outcome. Interaction between treatment status variables and covariates will be evaluated by inclusion of treatment by covariate interaction terms in the model. In a final set of analyses, we will compare the single end of study measure (change from baseline) for the outcome variable using a multivariable regression approach with treatment status as the primary independent variable, with missing values imputed using the method of Little and Rubin [83]. Analyses will be repeated for each of the clinical measures, both with and without a Bonferroni correction [84] for the multiple outcome variables. In other words, both the unadjusted and Bonferroni-corrected p-values will be obtained to evaluate sensitivity of conclusions to the "multiple comparisons" effect.

### Economic Analyses

A Markov-type economic model will be used to predict flow of funds and event rates over a 3-month and 12-month period for populations with depression based on outcomes and effectiveness data. The model will use actual event data reported by participants through survey at follow-up. The reason for including all costs is that it is sometimes difficult to distinguish depression-related from non-depression events. However, we will attempt to separate hospitalizations and office visits associated with a patient's depression problems from non-depression episodes of care. Another reason for including all costs is that depression is comorbid with many other conditions and the benefit of treatment may go beyond mental health costs. Thus, we will measure total cost savings and, based on patient response, separate depression-related costs from the cost offset. Capital costs will be annualized and depreciated over the life of the equipment, thus calculations for a 12-month period should be straightforward. Potential costs savings of Telepsychology will be based on sensitivity analysis of the fluctuations in the proportion of those appropriately treated with traditional Same-Room mental health care service delivery, compared to the actual proportion from those in the Telepsychology analysis.

Patient-oriented resource utilization data will be linked to participant's clinical data and a set of standard cost weights developed from archival VA billing data sources from patients with depression. The resources reported by the study participants (including medications) will be converted to dollar values using standard cost weights estimated from DSS administrative data. Average cost per day and average cost per admission will be calculated. Outpatient costs will include the average cost per clinic visit. Cost weights will be applied to the survey responses. To gain further insight into the variation in the resource utilization and costs of Telepsychology, sensitivity analyses will be performed. Sensitivity analysis involves systematically altering our assumptions about the costs and health effects of Telepsychology and noting the effect on the resulting cost/savings estimates. Two types of sensitivity analyses will be performed. The first set of calculations will be one-way sensitivity analyses. In one-way sensitivity analysis, the assumption about each parameter (eg, cost of telepsychology hardware) is varied over a reasonable range of values with all other parameters fixed. Finally, we will develop favorable and unfavorable scenarios for Telepsychology based on various assumptions including, but not limited to capital costs of telepsychology, technology "downtimes," and clinical outcomes. Because the specifics of these scenarios will depend on the nature of our findings, we cannot describe fully these analyses a priori. Nonetheless, these scenarios will reflect our understanding of the variation surrounding the cost estimates and the correlations among those parameters.

Cost data will be displayed in tables to highlight the extent to which costs cluster around videoconferencing technology, as well as patient-related utilization such as initial hospitalizations, outpatient visits, non-healthcare patient costs, medications and readmission(s). Costs will also be displayed graphically in a cumulative fashion, with cost on the horizontal axis and percent of patients with a specified or lower cost on the vertical axis. Finally costs will be displayed over time, with time on the x axis and mean cost on the y axis. Means and standard deviations of costs will be presented for each component of cost. "Trimmed" means and standard deviations of costs will be presented when 10% largest values and 10% smallest values are ignored. This way the summary statistics are less influenced by a small number of extreme outliers. Another possibility is to consider median as a measure of location and inter-quartile range as a measure of variance. However the usual or trimmed means and standard deviations seem more appropriate for this analysis because health care providers and payers may be concerned more about average cost, not median cost.

## Discussion

Study recruitment commenced in April 2007, with all follow-up assessments associated with the study expected to be completed by June 2010. As of October 2008, 171 participants have been screened and 111 have been randomized. Of the number randomized, 80 have completed Week 4 assessments, 72 have completed Week 8 assessments, 39 have completed Month 3 assessments, and 6 are due to complete Month 12 assessments.

Addressing a significant knowledge gap, this study is a prospective, randomized evaluation of clinical, process, and cost outcomes for a mental health intervention. The study will test the hypothesis that in-home Telepsychology service delivery will be equally effective as a traditional mode (Same-Room). If this study demonstrates telepsychology efficacy is equivalent to Same-Room service delivery, future research and program development will focus on bringing a wide range of specialized mental health services to the homes of veterans and community based outpatient clinics (eg, CBOCs, Veteran Centers) via videoconferencing technology. If Telepsychology non-inferiority and cost-effectiveness can be empirically established, research that examines a range of important system issues, including effectiveness outcomes and service delivery strategies to maximize the accessibility of mental health services for rural veterans can proceed. We anticipate that future research projects would include examination of telepsychology applications for providing individual and group mental health care to people with a range of other mental illnesses. Furthermore, we will also be able to evaluate the association between process and outcome measures in this study, which will have implications for future telepsychiatry efforts, as well as more general treatment outcome efforts with older veteran populations. Thus, while meeting the VA's mission to provide quality health care to all veterans, this project represents an important step in a programmatic line of research, including planned future VA HSR&D applications in mental health service delivery and care coordination to rural and underserved populations.

## Abbreviations

BAI: Beck's Anxiety Index; BATD: Behavioral Activation Treatment forDepression; BDI: Beck's Depression Index; CBOC: Community BasedOutpatient Clinic; CONSORT: Consolidated Standards of ReportingTrials; CPOSS: Charleston Psychiatric Outpatient Satisfaction Scale; DSM-IV: Diagnostic and Statistical Manual of Mental Disorders, 4th Edition; DSS: Decision Support System; GDS: Geriatric DepressionScale; HSR&D: Health Services Research and Development; HIPAA: Health Insurance Portability and Accountability Act; ICD-9: International Classification of Diseases, Ninth Revision; ITT: Intention to Treat; IRB: Institutional Review Board; MDD: MajorDepressive Disorder; OEF: Operation Enduring Freedom; OIF: OperationIraqi Freedom; POTS: Plain Old Telephone Service; RRM: RandomRegression Models; SCID: Structured Clinical Interviews for DSMDisorders; SF-36: Medical Outcomes Study Short Form – 36; VA: Department of Veterans Affairs; VAMC: Veteran Affairs Medical Center; VISN: Veteran Integrated Service Network.

## Competing interests

CL is the developer of the behavioral activation treatment for depression (BATD) used in this study. The other authors declare that they have no competing interests.

## Authors' contributions

LE, CF, RA, RK, PM and CL conceived of the study, obtained funding for the study, participated in its design and coordination and helped to draft the manuscript. LR drafted the manuscript. All authors read and approved the final manuscript.
